# Whole Blood DNA Methylation Changes Are Associated with Anti-TNF Drug Concentration in Patients with Crohn’s Disease

**DOI:** 10.1093/ecco-jcc/jjad133

**Published:** 2023-08-08

**Authors:** Simeng Lin, Eilis Hannon, Mark Reppell, Jeffrey F Waring, Nizar Smaoui, Valerie Pivorunas, Heath Guay, Neil Chanchlani, Claire Bewshea, Benjamin Y H Bai, Nicholas A Kennedy, James R Goodhand, Jonathan Mill, Tariq Ahmad

**Affiliations:** Gastroenterology, Royal Devon University Healthcare NHS Foundation Trust, Exeter, UK; Exeter Inflammatory Bowel Disease and Pharmacogenetics Research Group, University of Exeter, Exeter, UK; University of Exeter Medical School, Faculty of Health and Life Sciences, University of Exeter, Exeter, UK; Precision Medicine Immunology, AbbVie Inc., Chicago, IL, USA; Precision Medicine Immunology, AbbVie Inc., Chicago, IL, USA; Precision Medicine Immunology, AbbVie Inc., Chicago, IL, USA; Precision Medicine Immunology, AbbVie Inc., Chicago, IL, USA; Precision Medicine Immunology, AbbVie Inc., Chicago, IL, USA; Gastroenterology, Royal Devon University Healthcare NHS Foundation Trust, Exeter, UK; Exeter Inflammatory Bowel Disease and Pharmacogenetics Research Group, University of Exeter, Exeter, UK; Exeter Inflammatory Bowel Disease and Pharmacogenetics Research Group, University of Exeter, Exeter, UK; Genomics of Inflammation and Immunity Group, Wellcome Sanger Institute, Hinxton, UK; Postgraduate School of Life Sciences, University of Cambridge, Cambridge, UK; Gastroenterology, Royal Devon University Healthcare NHS Foundation Trust, Exeter, UK; Exeter Inflammatory Bowel Disease and Pharmacogenetics Research Group, University of Exeter, Exeter, UK; Gastroenterology, Royal Devon University Healthcare NHS Foundation Trust, Exeter, UK; Exeter Inflammatory Bowel Disease and Pharmacogenetics Research Group, University of Exeter, Exeter, UK; University of Exeter Medical School, Faculty of Health and Life Sciences, University of Exeter, Exeter, UK; Gastroenterology, Royal Devon University Healthcare NHS Foundation Trust, Exeter, UK; Exeter Inflammatory Bowel Disease and Pharmacogenetics Research Group, University of Exeter, Exeter, UK

**Keywords:** DNA methylation, epigenetics, anti-TNF, Crohn’s disease, IBD

## Abstract

**Background and Aims:**

Anti-tumour necrosis factor [TNF] treatment failure in patients with inflammatory bowel disease [IBD] is common and frequently related to low drug concentrations. In order to identify patients who may benefit from dose optimisation at the outset of anti-TNF therapy, we sought to define epigenetic biomarkers in whole blood at baseline associated with anti-TNF drug concentrations at week 14.

**Methods:**

DNA methylation from 1104 whole blood samples from 385 patients in the Personalised Anti-TNF Therapy in Crohn’s disease [PANTS] study were assessed using the Illumina EPIC Beadchip [v1.0] at baseline and weeks 14, 30, and 54. We compared DNA methylation profiles in anti-TNF-treated patients who experienced primary non-response at week 14 if they were assessed at subsequent time points and were not in remission at week 30 or 54 [infliximab *n* = 99, adalimumab n = 94], with patients who responded at week 14 and when assessed at subsequent time points were in remission at week 30 or 54 [infliximab *n* = 99, adalimumab *n* = 93].

**Results:**

Overall, between baseline and week 14, we observed 4999 differentially methylated positions [DMPs] annotated to 2376 genes following anti-TNF treatment. Pathway analysis identified 108 significant gene ontology terms enriched in biological processes related to immune system processes and responses. Epigenome-wide association [EWAS] analysis identified 323 DMPs annotated to 210 genes at baseline associated with higher anti-TNF drug concentrations at Week 14. Of these, 125 DMPs demonstrated shared associations with other common traits [proportion of shared CpGs compared with DMPs] including body mass index [23.2%], followed by C-reactive protein [CRP] [11.5%], smoking [7.4%], alcohol consumption per day [7.1%], and IBD type [6.8%]. EWAS of primary non-response to anti-TNF identified 20 DMPs that were associated with both anti-TNF drug concentration and primary non-response to anti-TNF with a strong correlation of the coefficients [Spearman’s rho = -0.94, *p* <0.001].

**Conclusion:**

Baseline DNA methylation profiles may be used as a predictor for anti-TNF drug concentration at week 14 to identify patients who may benefit from dose optimisation at the outset of anti-TNF therapy.

## 1. Introduction

Anti-tumour necrosis factor [TNF] therapies remain the most effective treatment to induce and maintain remission in patients with Crohn’s disease.^1,2^ Successful treatment leads to mucosal healing, reduced surgeries, and improvements in quality of life.^3^ Unfortunately, anti-TNF treatment failure is common, with a quarter of patients experiencing primary non-response and one-third of initial responders losing response by the end of the first year.^4^

In the Personalised Anti-TNF Therapy in Crohn’s Disease study [PANTS], loss of response was associated with the formation of anti-drug antibodies that were predicted by carriage of the HLA-DQA1*05 haplotype and mitigated by concomitant immunomodulator use, but the only modifiable factor associated with primary non-response at week 14 was low anti-TNF drug concentration.^5,6^ In this regard, early dose optimisation reportedly improves anti-TNF response rates.^7,8^ Whereas the biology of non-response is complex, an ability to predict primary non-response may inform treatment choice and identify individuals who may benefit from dose optimisation during induction therapy.

Heterogeneity of response to anti-TNF therapies has led to a drive to understand the molecular mechanisms underlying treatment failure in anti-TNF therapy. Increased mucosal expression of oncostatin M [*OSM*]^9,10^ and triggering receptor expressed on myeloid cells [*TREM-1*]^11,12^ have been identified as potential biomarkers predicting non-response to anti-TNF treatment. Drawing conclusions across studies is difficult however, due to differences in study design, improvements in experimental and computational methods through time, and critically, confounding by cellular heterogeneity with contradictory results between whole blood and intestinal biopsies.^13^ Clinical translation of tissue biomarkers has also further been limited by accessibility and processing costs.^14^

DNA methylation, an epigenetic modification to DNA, can influence gene expression via disruption of transcription factor binding and recruitment of methyl-binding proteins that initiate chromatin compaction and gene silencing.^15,16^ Despite being traditionally regarded as a mechanism of transcriptional repression, DNA methylation is actually associated with both increased and decreased gene expression^17^ and other genomic functions including alternative splicing and promoter usage.^18^ DNA methylation can be influenced by both genetic^19^ and environmental factors,^20^ changing with age^21^ and exposures such as cigarette smoking.^22^ The development of standardised assays for quantifying DNA methylation across the genome, at single-base resolution in large numbers of samples, has enabled researchers to perform epigenome-wide association studies [EWAS] aimed at identifying methylomic variation associated with exposures and traits.^23^ EWAS analyses are inherently more complex to design and interpret than genetic association studies; the dynamic nature of epigenetic processes means that a range of potentially important confounding factors [including tissue or cell type, age, sex, and lifestyle exposures] need to be considered in between-group comparisons.^24^

Previous studies of DNA methylation using whole blood or individual purified cell types have identified differentially methylated positions [DMPs] between patients with active and inactive IBD and healthy controls, and for the classification of different IBD sub-types.^25–27^ Pharmacoepigenomics is the application of epigenetics to understand inter-individual differences in response to therapeutic drugs.^28^ DNA methylation sites from whole blood have been identified as effective biomarkers predicting treatment response to methotrexate and anti-TNF in patients with rheumatoid arthritis.^29–31^

In this study we used a powerful intra-individual study design to identify changes in DNA methylation associated with anti-TNF drug treatment, profiling 385 patients at baseline and weeks 14, 30, and 54 post treatment initiation. In order to identify patients who may benefit from dose optimisation at the outset of anti-TNF therapy, we also sought to define epigenetic biomarkers in whole blood at baseline associated with anti-TNF drug concentrations at week 14. We identify widespread differences in DNA methylation induced by anti-TNF drug treatment, and show that baseline DNA methylation profiles can predict anti-TNF drug concentration at Week 14.

## 2. Methods

### 2.1. Study design

The PANTS study is a UK-wide, multicentre, prospective, observational cohort reporting the treatment failure rates of the anti-TNF drugs infliximab, originator (Remicade [Merck Sharp & Dohme, UK]) and biosimilar CTP13 [Celltrion, Incheon, South Korea]), and adalimumab (Humira [AbbVie, Cambridge, MA]), in 1610 anti–TNF-naïve patients with Crohn’s disease [Supplementary Table 1].

Patients were recruited between February 2013 and June 2016 at the time of first anti-TNF exposure, and were studied for 12 months or until drug withdrawal. Eligible patients were aged ≥6 years, with objective evidence of active luminal Crohn’s disease involving the colon and/or small intestine. Exclusion criteria included prior exposure to, or contraindications for, the use of anti-TNF therapy. The choice of anti-TNF was at the discretion of the treating physician and prescribed according to the licensed dosing schedule. Study visits were scheduled at first dose, week 14, and at weeks 30 and 54. Additional visits were planned for infliximab-treated patients at each infusion and for both groups at treatment failure or exit. Treatment failure endpoints were primary non-response at week 14, non-remission at week 54, and adverse events leading to drug withdrawal.

We used composite endpoints using the Harvey–Bradshaw Index [HBI] in adults and the short Paediatric Crohn’s Disease Activity Index [sPCDAI] in children, corticosteroid use, and CRP to define primary non-response [Supplementary Figure 1]. Remission was defined as CRP of ≤3 mg/L and HBI of ≤4 points [sPCDAI ≤15 in children], without corticosteroid therapy or exit for treatment failure.

Variables recorded at baseline were patient demographics [age, sex, ethnicity, comorbidities, height, weight, and smoking status] and IBD phenotype and its treatments [age at diagnosis, disease duration, Montreal classification, prior medical and drug history, and previous Crohn’s disease-related surgeries]. At every visit, disease activity score, weight, current therapy, and adverse events were recorded. Blood and stool samples were collected at each visit and processed through the central laboratory at the Royal Devon & Exeter NHS Foundation Trust [https://www.exeterlaboratory.com/] for haemoglobin, white cell count, platelets, serum albumin, CRP, anti-TNF drug and anti-drug antibody concentrations, and faecal calprotectin. Matched genetic data were also collected, using the methods described previously.^6^

### 2.2. DNA methylation processing

Genomic DNA was extracted from peripheral whole blood using the Qiagen Qiasymphony DNA DSP midi kit [Qiagen, CA, USA]. Following sodium bisulphite conversion with the Zymo Research EZ-DNA Methylation kit [Zymo Research, CA, USA], DNA methylation was quantified across the genome using the Illumina Infinium HumanMethylationEPIC [EPIC] BeadChip v1.0 [Illumina, CA, USA]. To negate any methodological batch effects, individuals were randomised across experimental batches and samples from the same individual were processed together across all experimental stages.

### 2.3. Data pre-processing and quality control checks

Raw Illumina EPIC data were imported into R [version 3.6.0] using the *bigmelon* package [v1.12.0].^32^ Quality control [QC] checks were performed using the *bigmelon* [v1.12.0]^32^ and *minfi* [v1.32.0]^33^ R packages. They included the following steps: we first removed samples by 1] checking median methylated and unmethylated signal intensities and excluding samples with low intensities [<500] [three samples excluded]; 2] assessing bisulphite conversion efficacy of each sample and excluding samples with a conversion rate <80% [nine samples excluded]; 3] using the 59 single nucleotide polymorphism [SNP] probes present on the EPIC array to confirm all matched samples from the same individual were genetically identical and to check for sample switches or duplications [12 samples excluded]; 4] comparing intensity values from probes located on the X and Y chromosomes with autosomes to identify sex mismatches [10 samples excluded]; 5] visually inspecting the first six principal components and excluding outliers [none identified]; and 6] using the pfilter function in the *bigmelon* [v1.12.0] package to exclude samples where >1% of probes had a detection *p*-value >0.05 [none identified]. We subsequently removed probes by 7] using the pfilter function in the *bigmelon* [v1.12.0] package to exclude probes with a bead count <3 or 1% of samples with a detection *p*-value >0.05 [8313 probes]; and 8] removal of cross-hybridising probes, SNP probes, and probes affected by common SNPs [73 239 probes]. Following QC, quantile normalisation was carried out and 784 105 probes were taken forwards for analysis after exclusion of probes on the Y chromosome.

### 2.4. Sample size and statistical methods

Sample size calculations from the whole PANTS cohort have been reported previously.^5^ For this analysis, we selected whole blood samples from a subset of 385 participants treated with infliximab and adalimumab, aged >16 years, with a baseline CRP ≥4 mg/L and/or calprotectin >100 µg/g, who experienced primary non-response at week 14, and if they were assessed at later time points, were in non-remission at weeks 30 or 54 [*n* = 99 and 94, respectively]. We selected an equal number of participants as a comparator group, who were classified with primary response at week 14 and, if they were assessed at later time points, in remission [*n* = 99 and 93, respectively] for DNA methylation profiling.

Statistical analyses were undertaken in R 4.1.3 [R Foundation for Statistical Computing, Vienna, Austria]. We included patients with missing clinical variables in analyses for which they had the necessary data, and have specified the sample size for each variable. Continuous data are reported as median and interquartile range, and discrete data as numbers and percentages. Fisher’s exact and Mann–Whitney U tests were used to identify differences in baseline characteristics between infliximab-treated and adalimumab-treated patients. Comparative tests were two-tailed, and a *p*-value <0.05 was considered significant unless otherwise stated.

DNA methylation was analysed using beta values, the ratio of methylated intensity to the overall intensity at each CpG site, which represents the proportion of methylation at each site. Because they influence methylation, a priori we sought to define DNA methylation changes through the course of the study due to ageing, cigarette smoking, and cell composition. Smoking scores were calculated using a weighted sum approach based on previously published smoking-associated methylation probes.^34^ Epigenetic age was predicted using 353 CpG sites as described by Horvath.^21^ Individual cell proportions of CD4+T cells, CD8+T cells, B cells, granulocytes, and monocytes in each whole blood sample were estimated using the Houseman reference-based algorithm implemented with functions in the *minfi* [v1.32.0]^33^ package. Linear mixed effects models, including time on anti-TNF [study visits in weeks] as a fixed effect and modelling individual participants with a random intercept, were used to determine if epigenetic age, smoking behaviour, or cell composition were associated with anti-TNF treatment.

EWAS analyses of anti-TNF treatment, anti-TNF drug concentration, primary non-response, and development of anti-drug antibodies stratified by carriage of the HLA-DQA1*05 variant were conducted using linear mixed effects models in this within-subject study, where anti-TNF type and cell proportions were adjusted for as fixed effects and a random effect [random intercept] was used to capture the individual-level effects. Study visits in weeks, reflecting the duration of anti-TNF treatment, were included as an interaction term in the model, allowing all samples at different time points to be included. Patients treated with infliximab and adalimumab were analysed together to increase the power to detect shared effects. An empirically-derived *p-*value <9 x 10^-8^ was considered significant to control for multiple testing.^35^ To determine if the significant DMPs identified in whole blood could be attributed to variation driven by specific blood cell types, we compared the DMPs with previously described characteristic scores,^36^ which represents the extent to which the variation in DNA methylation in each individual blood cell type relates to the variation measured in whole blood at each CpG site. Pathway analysis with annotations to gene ontology [GO] terms was performed using the *gometh []* function in *missMethyl* [v1.28.0]^37^ package, which controls for bias arising due to multiple genes being annotated to a single CpG and multiple CpGs annotated to a single gene. DMPs were searched in the EWAS catalogue^38^ [http://www.ewascatalog.org/, assessed on December 15, 2022] to look for associations with other common traits. A false-discovery rate [FDR] of <0.05 was considered significant for pathway analysis and associations in the EWAS catalogue. We sought overlapping DMPs associated with drug levels and primary non-response, and correlation of coefficients was determined using Spearman’s test. Gene expression changes of genes annotated to DMPs at baseline associated with primary non-response were compared between those who responded and those who did not. Detailed methods and results can be found in a separate manuscript.^39^ A Bonferroni correction for the number of genes compared [*n* = 28] was applied to the *p*-values, and an adjusted *p*-value of <0.05 was considered significant.

### 2.5. Ethical approval and role of the funding source

The sponsor of the study was the Royal Devon and Exeter NHS Foundation Trust. The South West Research Ethics Committee approved the study [REC Reference: 12/SW/0323] in January 2013. The funders of this study had no role in study design, data collection, data analysis, data interpretation, or writing of the report. The corresponding author had full access to all the data in the study and had final responsibility for the decision to submit for publication.

## 3. Results

### 3.1. Summary of PANTS DNA methylation dataset

DNA methylation was quantified across the genome in 1104 whole blood DNA samples from 385 individuals across four study visits [baseline, week 14, week 30, week 54] from the PANTS cohort. Following a standard quality control pipeline [see Methods], our final dataset included 784 105 DNA methylation sites quantified in 1062 samples from 385 participants [Supplementary Figure 1]; 87 participants provided samples at all four study visits, and the median number of samples per participant was three (interquartile range [IQR] 2–3) [Supplementary Table 2].

Overall, 51.7% [199/385] of participants were female, with a median age of 35.7 years [IQR 26.3–50.3], 21.2% [81/382] of participants were current smokers, and 30.6% [117/382] were former smokers. The median disease duration was 2.2 years [IQR 0.6–9.6], and 50.9% [196/385] and 35.3% [136/385] of participants were treated with a concomitant immunomodulator and steroid at baseline, respectively. In total, 51.4% [198/385] of participants were treated with infliximab and 48.6% [187/385] with adalimumab [Table 1]. Median infliximab [3.30 mg/L vs 8.09 mg/L, *p* <0.001] and adalimumab [7.70 mg/L vs 13.35 mg/L, *p* <0.001] drug concentrations at week 14 were lower in patients who experienced primary non-response, as previously observed in the wider cohort^5^ [Supplementary Figure 2].

**Table 1.** Characteristics at baseline of participants stratified by type of anti-TNF.

Variable	Level	Adalimumab	Infliximab	Overall	*p*
*n*		187	198	385	
Age at first dose		37.19 [26.56–51.15]	35.25 [25.57–49.49]	35.69 [26.34–50.26]	0.466
Sex	Female	47.06% [88/187]	56.06% [111/198]	51.69% [199/385]	0.083
	Male	52.94% [99/187]	43.94% [87/198]	48.31% [186/385]	
Ethnicity	White	94.12% [176/187]	94.95% [188/198]	94.55% [364/385]	0.933
	South Asian	2.14% [4/187]	2.02% [4/198]	2.08% [8/385]	
	Other	3.74% [7/187]	3.03% [6/198]	3.38% [13/385]	
Smoking history	Current	17.20% [32/186]	25.00% [49/196]	21.20% [81/382]	0.163
	Ex	33.33% [62/186]	28.06% [55/196]	30.63% [117/382]	
	Never	49.46% [92/186]	46.94% [92/196]	48.17% [184/382]	
Disease duration [years]		2.80 [0.61–9.53]	2.08 [0.53–9.52]	2.17 [0.56–9.53]	0.483
Montreal location classification	L1	31.52% [58/184]	29.44% [58/197]	30.45% [116/381]	0.956
	L2	25.54% [47/184]	26.90% [53/197]	26.25% [100/381]	
	L3	42.39% [78/184]	43.15% [85/197]	42.78% [163/381]	
	L4	0.54% [1/184]	0.51% [1/197]	0.52% [2/381]	
Montreal behaviour classification	B1	59.24% [109/184]	63.96% [126/197]	61.68% [235/381]	**0.035**
	B2	35.33% [65/184]	25.38% [50/197]	30.18% [115/381]	
	B3	5.43% [10/184]	10.66% [21/197]	8.14% [31/381]	
Immunomodulator use at baseline	TRUE	52.94% [99/187]	48.99% [97/198]	50.91% [196/385]	0.476
Steroid use at baseline	TRUE	29.41% [55/187]	40.91% [81/198]	35.32% [136/385]	**0.019**
C-reactive protein [mg/L]		8.00 [4.00–19.50]	12.00 [5.00–32.00]	10.00 [5.00–24.00]	**0.013**
HBI score		5.00 [3.00–8.00]	6.00 [3.00–9.50]	5.00 [3.00–9.00]	0.256
Faecal calprotectin [ug/g]		307.00 [159.50–599.00]	481.00 [251.00–881.50]	365.00 [188.00–726.00]	**0.001**
Haemoglobin [g/L]		129.50 [120.00–140.00]	125.00 [112.00–135.00]	128.00 [116.00–137.00]	**0.002**
White cell count [x 10^9^ cells per L]		7.90 [6.20–10.20]	8.56 [6.60–10.70]	8.23 [6.40–10.40]	0.166

Characteristics which are significantly different between the anti-TNFs are highlighted in bold.

TNF, tumour necrosis factor; HBI, Harvey–Bradshaw Index.

### 3.2. Anti-TNF treatment is associated with altered blood cell proportions using measures derived from DNA methylation data

A number of robust statistical classifiers have been developed to derive estimates of environmental exposures such as tobacco smoking,^34^ biological age,^21^ and the proportion of different blood cell types^40^ from whole blood DNA methylation data.

As expected, current and former tobacco smokers had a higher DNA methylation-derived smoking score at baseline (former smokers 0.2 [IQR -2.0–4.1], *p* <0.001 and current smokers 6.6 [IQR 3.3–9.5], *p* <0.001) when compared with never smokers (-2.3 [IQR -3.8 – -1.0]). Over the duration of the study, DNA methylation smoking score increased [effect size per week 0.019, *p* <0.001]. When compared with current smokers, the trajectory of DNA methylation smoking score changed significantly in former [effect size per week -0.010, *p* = 0.003], but not current smokers [effect size per week -0.004, *p* = 0.262) [Supplementary Figure 3]. When stratified by response to anti-TNF treatment, following anti-TNF treatment, there was no difference in the trajectory of smoking scores of current smokers [effect size per week -0.006, *p* = 0.433] or former smokers [effect size per week -0.0001, *p* = 0.986] between those who experienced primary non-response compared with those who did not.

The epigenetic age of participants measured using the Horvath multi-tissue clock was highly correlated with chronological age of participants at study entry [r = 0.95, *p* <0.001]. Over the course of the study following anti-TNF treatment, epigenetic age changed with time [effect size per week 0.002, *p* <0.001]. When stratified by response to anti-TNF treatment, however, there was no difference in the trajectory of change in epigenetic age [effect size per week 0.003, *p* = 0.71] [Supplementary Figure 4].

**Figure 1.**
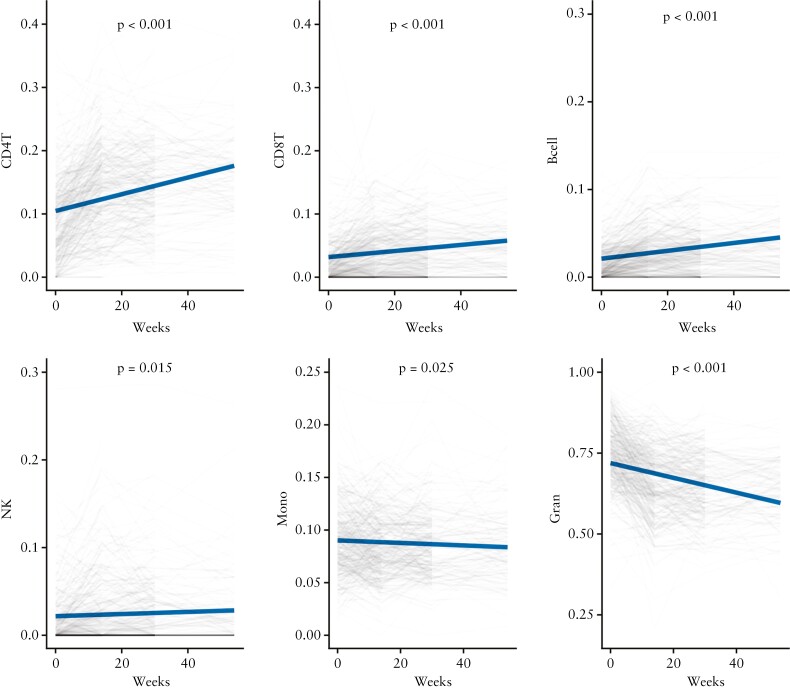
Change in derived cell proportions following treatment with anti-TNF. Predicted derived cell proportions over time estimated from the regression analysis is represented in solid blue lines, and observed cell proportions in faded lines; *p*-value represents the change in individual cell proportions over time. TNF, tumour necrosis factor.

To understand the immune cell changes following anti-TNF treatment, cell proportion estimates were derived from DNA methylation data. Over time, following anti-TNF treatment, there was a significant increase in the derived proportions of CD4 T cells [effect size per week 0.0013, *p* <0.001], CD8 T cells [effect size per week 0.0005, *p* <0.001], B cells [effect size per week 0.0004, *p* <0.001], and NK cells [effect size per week 0.0001, *p* = 0.015] [Figure 1]. In contrast, the proportion of monocytes [effect size per week -0.0001, *p* = 0.025] and granulocytes [effect size per week -0.0023, *p* <0.001] decreased significantly. In patients who experienced primary non-response, the increase in proportion of B cells [effect size per week -0.0002, *p* <0.001] and CD4 T cells [effect size per week -0.0004, *p* = 0.048] were less marked over time when compared with those who responded. There was no difference in the change of proportion of granulocytes [effect size per week 0.0002, *p* = 0.571].

### 3.4. Changes in biological processes of the immune pathways occur following anti-TNF treatment

**Figure 2.**
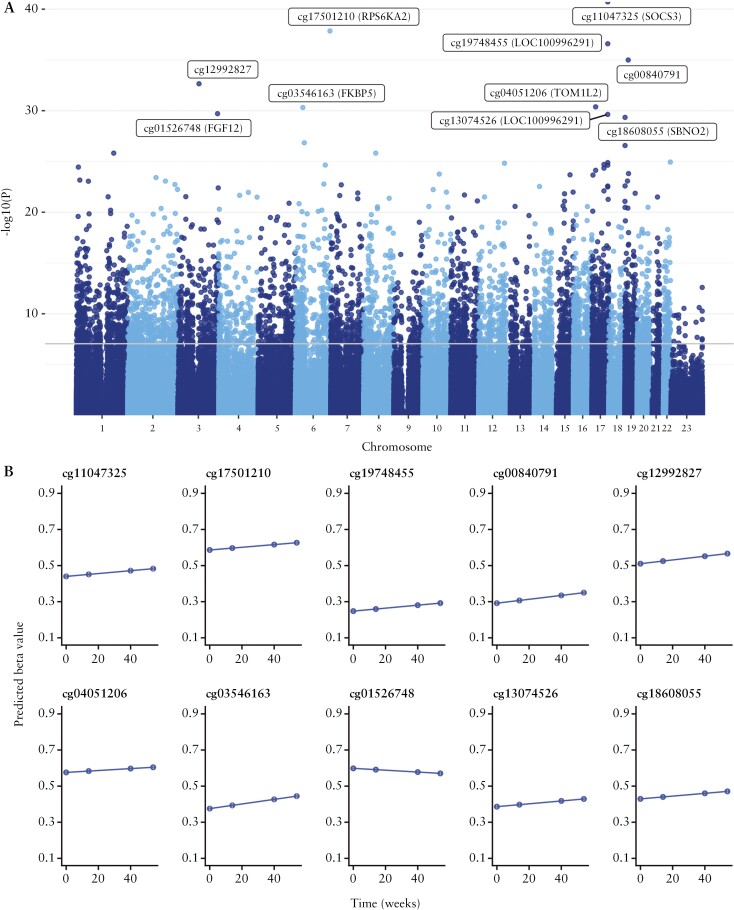
CpG sites associated with change over time following anti-TNF treatment regardless of treatment outcome. A] Manhattan plot of CpG sites associated with change over time following anti-TNF treatment regardless of treatment outcome. The top 10 differentially methylated positions with annotations are labelled in the plot. The grey horizontal line represents the significant *p-*value threshold of 9 x 10^-8^. B] Predicted beta values of the top 10 differentially methylated positions and its change over time following anti-TNF treatment. TNF, tumour necrosis factor.

Across all patients, 4999 DMPs [*p* <9 x 10^-8^] with 3504 DMPs annotated to 2376 unique genes were associated with anti-TNF treatment [infliximab or adalimumab] regardless of response [Table 2, Figure 2]. These DMPs were significantly enriched for sites becoming hypomethylated over time (63.5% [3176/4999], *p* <0.001). Of treatment-associated DMPs annotated to genes [n = 3504 [70.1%]), the majority were located in the gene body (67.1% [2351/3504]) [Supplementary Figure 5], representing a significant enrichment compared with the background distribution of probes on the EPIC array [67.1% vs 29.5%, *p* <0.001]. The top-ranked DMP associated with anti-TNF treatment was cg11047325 annotated to the *SOCS3* gene, involved in the negative regulation of the JAK-STAT pathway and thought to play a role in modulating the outcome of infections and autoimmune diseases^41^ [effect size per week 0.0008, *p* = 1.91 x 10^-41^].

**Table 2.** Top 10 differentially methylated positions associated with anti-TNF treatment over time.

CpG	Chromosome	Position	Relation to island	Gene name	Gene group	Coefficient [per week]	*p*-value
cg11047325	17	76354934	Island	SOCS3	Body	0.0008	1.91E-41
cg17501210	6	166970252	OpenSea	RPS6KA2	Body	0.0007	1.40E-38
cg19748455	17	76274856	OpenSea	LOC100996291	TSS1500	0.0008	2.55E-37
cg00840791	19	16453259	OpenSea			0.0011	1.02E-35
cg12992827	3	101901234	OpenSea			0.0010	2.23E-33
cg04051206	17	17750855	OpenSea	TOM1L2	3’UTR	0.0005	4.19E-31
cg03546163	6	35654363	N_Shore	FKBP5	5’UTR	0.0012	4.87E-31
cg01526748	3	191930926	OpenSea	FGF12	Body	-0.0005	1.97E-30
cg13074526	17	76274743	OpenSea	LOC100996291	TSS200	0.0008	2.32E-30
cg18608055	19	1130866	OpenSea	SBNO2	Body	0.0008	4.57E-30

TNF, tumour necrosis factor.

Of the 4999 DMPs, variation in only five DMPs could be attributed to specific blood cell types: four DMPs associated with B cells and one DMP associated with CD8 T cells. The remaining DMPs could not be confidently assigned to a specific cell type. Gene ontology [GO] analysis of genes annotated to treatment-associated DMPs identified 108 significant biological pathways [Supplementary Table 3], further implicating the immune response (immune system process [GO: 0002376, FDR <0.001], immune response [GO: 0006955, FDR <0.001], and immune system development [GO:0002520, FDR <0.001]) alongside pathways related to blood cell differentiation (haematopoietic or lymphoid organ development [GO:0048534, FDR <0.001] and haemopoiesis [GO: 0030097, FDR <0.001]) [Supplementary Figure 6].

### 3.4. DNA methylation in infliximab- and adalimumab treated patients

Next, we performed an epigenome-wide association study [EWAS] to identify any DMPs associated with anti-TNF treatment type. Overall, there were no significant DMPs at baseline between anti-TNF–naïve Crohn’s disease patients who were subsequently treated with infliximab or adalimumab. Irrespective of primary non-response status, we observed 13 DMPs annotated to nine genes with significantly different trajectories following treatment with infliximab compared with adalimumab. The top-ranked DMPs between treatments included cg03446165 [annotated to *MMP25*] [effect size per week -0.0004, *p* = 6.78 x 10^-10^], cg12229367 [effect size per week -0.0003, *p* = 1.54 x 10^-9^], and cg04790662 [annotated to *PAG1*] [effect size per week -0.0005, *p* = 2.82 x 10^-9^] [Supplementary Table 4]. When stratified by response to anti-TNF, no CpG sites were significantly associated with primary non-response to infliximab- compared with adalimumab-treated individuals either at baseline or over time.

### 3.5. DNA methylation differences at baseline are associated with anti-TNF drug concentration following treatment and primary non-response

**Figure 3.**
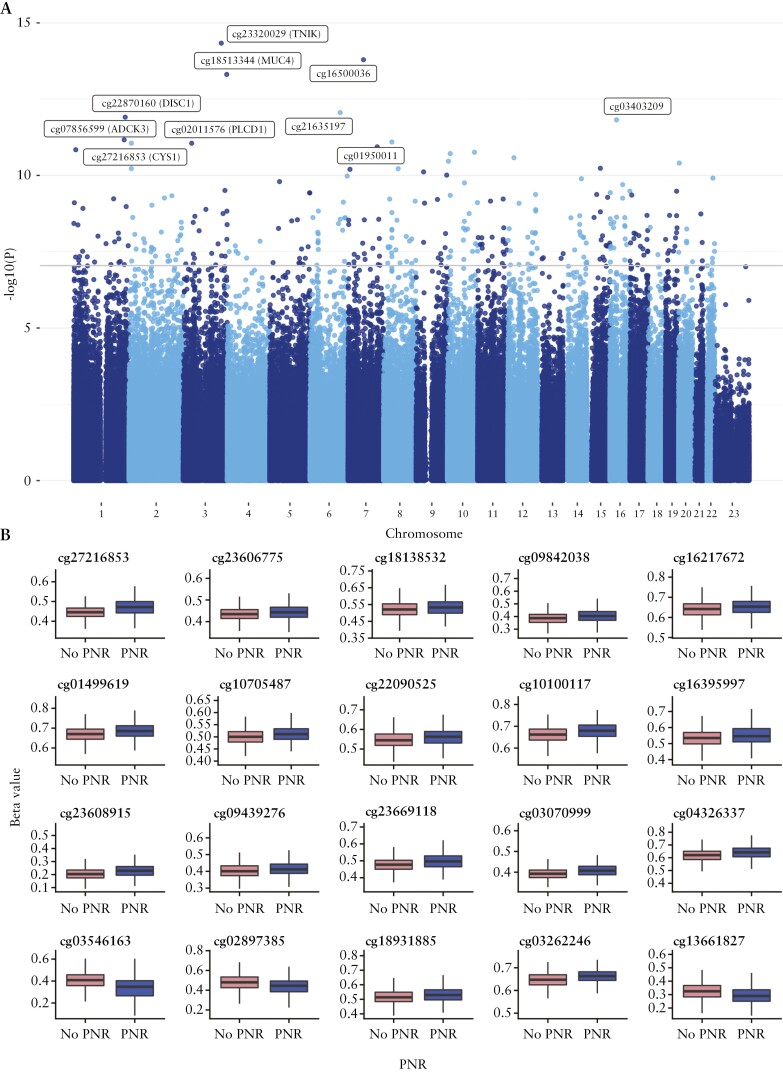
CpG sites at baseline associated with anti-TNF drug concentration. A] Manhattan plot of CpG sites at baseline associated with anti-TNF drug concentration at week 14. The top 10 CpG sites with their associated gene annotations are labelled in brackets. The grey horizontal line represents the significant *p*-value threshold of 9 x 10^-8^. B] Beta methylation values at baseline of the top 20 CpG sites associated with both anti-TNF drug concentration at week 14 and primary non-response. TNF, tumour necrosis factor.

We sought to determine if DNA methylation differences at baseline prior to the start of anti-TNF treatment were associated with anti-TNF drug concentrations at week 14. We identified 323 DMPs, with 227 DMPs annotated to 210 genes at baseline associated with anti-TNF drug concentrations at week 14 [Table 3, Figure 3]. The top ranked DMP was cg23320029 annotated to the *TNIK* gene [effect size 0.0555, *p* = 4.62 x 10^-15^], encoding the TRAF2 and NCK-interacting kinase, a key regulator in the Wnt signalling pathway implicated in the modulation of immune response during inflammation.^42^ Of the 323 DMPs, variations in 26 DMPs were found to be driven by specific blood cell types; 13 DMPs were associated with B cells, six with granulocytes, four with CD8 T cells, and three with monocytes. GO analysis of genes annotated to DMPs associated with anti-TNF drug concentration at week 14, however, did not identify any FDR [FDR <0.05] significant pathways, likely reflecting the inadequate number of genes to perform a meaningful pathway analysis.

**Table 3.** Top 10 differentially methylated positions at baseline associated with anti-TNF drug concentration at week 14

CpG	Chromosome	Position	Relation to island	Gene name	Gene group	Coefficient [per week]	*p*-value
cg23320029	3	171004750	OpenSea	TNIK	Body	0.0555	4.62E-15
cg16500036	7	68983906	OpenSea			-0.0409	1.61E-14
cg18513344	3	195531298	OpenSea	MUC4	Body	0.0299	4.90E-14
cg21635197	6	135405808	OpenSea			0.0389	8.71E-13
cg22870160	1	231830793	OpenSea	DISC1	Body	-0.0376	1.23E-12
cg03403209	16	29666641	OpenSea			-0.0247	1.51E-12
cg07856599	1	227151843	OpenSea	ADCK3	Body	-0.0411	6.82E-12
cg01950011	8	37396511	OpenSea			-0.0213	8.00E-12
cg27216853	2	10205672	OpenSea	CYS1	Body	-0.0371	8.79E-12
cg02011576	3	38060265	OpenSea	PLCD1	Body	-0.0387	8.82E-12

TNF, tumour necrosis factor.

We intersected the list of DMPs predicting anti-TNF drug concentration following treatment with the EWAS catalogue,^38^ to identify overlaps with DNA methylation differences associated with other traits and diseases, and finding that 125 [38.7%] DMPs have been previously associated with other common traits [Supplementary Table 5]. The most common shared association [proportion of shared CpGs compared with DMPs] was with an EWAS of body mass index [23.2%], followed by CRP [11.5%], smoking [7.4%], alcohol consumption per day [7.1%], and IBD type [6.8%]. The associations with these common traits all had a direction of effect opposite to anti-TNF drug concentration in our cohort; CpG sites associated with a higher BMI and increased CRP were associated with lower anti-TNF drug concentrations, in keeping with the known associations with anti-TNF drug concentration and treatment outcomes.^5^

**Figure 4.**
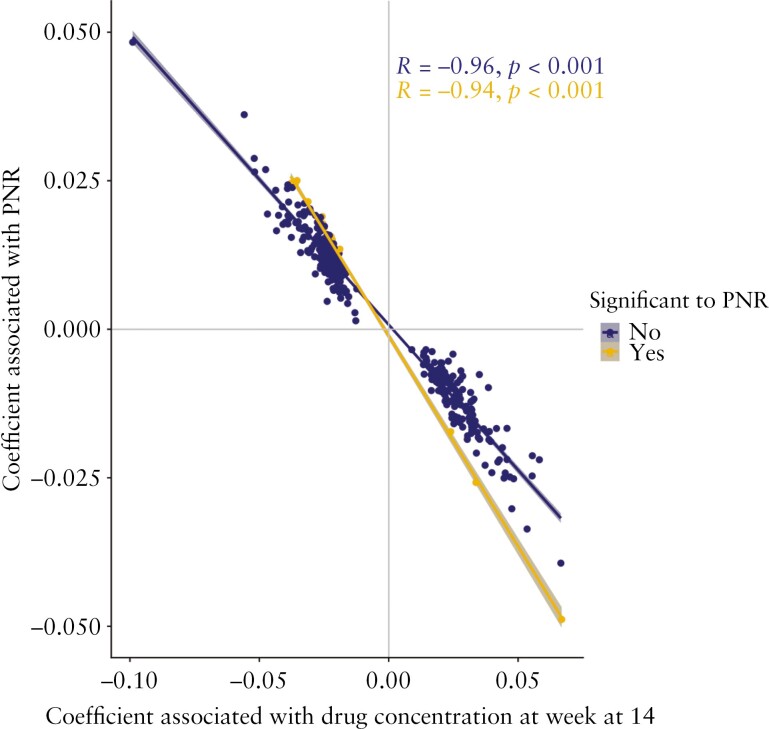
Coefficients of DMPs associated with anti-TNF drug concentration at week 14 and primary non-response. Coefficients represent the beta values of each CpG from linear mixed effects model to each outcome. Spearman’s rho correlation of the coefficients calculated for those that were associated with both anti-TNF drug concentration and primary non-response, and the remaining that were only significant to anti-TNF drug concentration. TNF, tumour necrosis factor; PNR, primary non-response; DMP, differentially methylated positions.

To understand if there was a relationship between anti-TNF drug concentration at week 14 and anti-TNF treatment response, we performed an EWAS of primary non-response to anti-TNF, and identified 48 DMPs annotated to 36 genes at baseline. Two DMPs each were annotated to the genes *CYS1*, *UPF2*, *LPAR5*, and *WDR8*. Of the 48 DMPs, 20 were associated with both anti-TNF drug concentration and primary non-response to anti-TNF [Supplementary Table 6]. These DMPs included cg27216853 [*CYS1*] [effect size to drug concentration -0.0371 vs effect size to primary non-response 0.0245], cg23606775 [*CLSTN1*] [-0.0220 vs 0.0133], and cg18138532 [*UPF2*] [-0.0273 vs 0.0157]. Overall, there was a strong correlation of the coefficients [Spearman’s rho = -0.94, *p* <0.001] [Figure 4], suggesting a relationship between DMPs associated with lower anti-TNF drug concentration and primary non-response.

Of the 36 genes annotated to DMPs at baseline associated with primary non-response, 27 had corresponding gene expression data^39^ [Supplementary Table 7]. Gene expression change in *FKBP5* was nominally significant between those who experienced response compared with those who experienced primary non-response to anti-TNF [log 2-fold change = -0.3551, adjusted *p*-value = 0.042]. No significant difference in gene expression was found in the rest of the 26 genes.

Finally, we sought to determine the relationship between DNA methylation and the HLA-DQA1*05 haplotype, known to be associated with development of anti-drug antibodies to anti-TNF treatment which can influence anti-TNF drug concentration. DNA methylation at eight DMPs was associated with carriage of the HLA-DQA1*05 haplotype [Supplementary Table 8], indicating that the locus is a DNA methylation quantitative trait locus [mQTL]; however, we did not identify any DMPs associated with the development of anti-TNF anti-drug antibodies, regardless of carriage of the HLA-DQA1*05 haplotype.

### 3.6. Longitudinal changes in DNA methylation differ in patients with primary non-response to anti-TNF treatment

Following anti-TNF treatment, intra-individual changes in DNA methylation were significantly different between those who experienced primary non-response to anti-TNF compared with those who did not, at five DMPs. These sites were cg07839457 [annotated to *NLRC5*] [effect size per week -0.0007, *p* = 1.92 x 10^-13^], cg11047325 [annotated to *SOCS3*] [-0.0007, *p* = 3.70 x 10^-11^], cg15022400 [annotated to *TRIM69*] [-0.0003, *p* = 3.39 x 10^-9^], cg25867318 [annotated to *STAT3*] [-0.0004, *p* = 6.06 x 10^-8^], and cg08950751 [annotated to *AIP*] [-0.0003, *p* = 6.82 x 10^-8^] [Supplementary Table 9]. The top ranked DMP following anti-TNF treatment, cg11047325 annotated to *SOCS3*, involved in regulation of the JAK-STAT pathway, was again identified.

## 4. Discussion

### 4.1. Key results

In whole blood, we observed almost 5000 DMPs annotated to >2000 genes that are associated with anti-TNF therapy, with the genes annotated to these sites being enriched for biological processes related to immune system processes. At week 14, 323 DMPs annotated to 210 genes were associated with anti-TNF drug concentration, and we observed an overlap between differentially methylated positions associated with drug concentrations and primary non-response.

### 4.2. Interpretation

It is perhaps unsurprising that treatment with the anti-TNF monoclonal antibodies infliximab and adalimumab led to a significant number of differentially methylated positions across multiple genes that were enriched in immune system pathways. Further, our findings of differential methylation in CpGs annotated to *SOCS3* and *STAT3*, thought to be involved in regulation of the JAK-STAT pathway which has a role in the inflammatory response of patients with IBD,^43^ provides additional insights into the mechanistic action of anti-TNF therapy in patients with IBD. Overall, however, only 13 DMPs were found when comparing infliximab- and adalimumab-treated patients over time, as compared with 4999 DMPs common to both anti-TNF treatments that changed over time, suggesting that there is an anti-TNF treatment class effect and that both drugs exert a similar effect upon levels of DNA methylation. A similar conclusion was made from a study of patients with rheumatoid arthritis treated with several different anti-TNFs including adalimumab, certolizumab, etanercept, golimumab, and infliximab, with no DMPs identified between different anti-TNF subtypes.^44^The immune cell changes and intracellular signalling pathways in peripheral blood and intestinal tissue following treatment with anti-TNF in patients with IBD is still unclear.^45^ Unlike a previous study of 14 patients with IBD,^46^ following anti-TNF treatment we did not observe a change in derived granulocyte proportions between non-responders and responders, but noted differences in B cells and CD4 T cells. Derived cell proportions at baseline were, however, not useful as a biomarker of anti-TNF non-response.

About a third [38.7%] of the DMPs associated with low drug concentrations were linked to other common traits, including body mass index, smoking, and CRP, that in the PANTS cohort were associated with drug concentration and anti-TNF treatment failure.^5^ It is plausible that these DMPs could be used as blood biomarkers independent of clinical traits, to predict inter-individual variability in anti-TNF drug concentration. If replicated, our epigenetic biomarker might allow pre-treatment identification of these at-risk individuals who might then be subjected to more intensive, therapeutic drug monitoring-driven, dosing strategies, allowing early effective anti-TNF dose prescribing. Our findings that the DMPs were enriched in gene bodies may suggest a more complex mechanism, apart from gene transcription, in their role underlying anti-TNF treatment response. The role of gene body methylation is still widely debated, and whereas they have been associated with the regulation of gene expression, they have a more complex role in suppressing aberrant gene transcription and regulating alternative splicing.^47^ With the advancement of single-cell sequencing technologies, the study of specific cell types in both disease-specific intestinal tissue^48^ and peripheral whole blood,^49^ perhaps based on our data focusing on the role of B- and CD4^+^ T cells, may provide further insights into the molecular mechanisms underlying anti-TNF treatment failure.

There was a strong correlation of effect between DMPs associated with lower drug concentration at week 14 and primary non-response, but the modest effect sizes mean that these markers are unlikely to be useful as a diagnostic predictor of primary non-response in individual patients. Why primary non-response is so difficult to predict in patients with IBD is unclear. Few of the so-called precision medicine biomarkers to facilitate the right drug, to the right patient, at the right time, have been replicated or translated to clinical care. There are a number of possible reasons for this. First, there are the challenges of defining primary non-response in the absence of endoscopic outcome data. In the PANTS study, we used a pragmatic, composite outcome closely linked to routine clinical care, which included patient symptoms assessed using validated severity scores and serum CRP. However, there is poor concordance between symptoms and biomarkers and mucosal inflammation. Patients with Crohn’s disease may also complain of symptoms suggestive of active disease because of overlapping irritable bowel syndrome, bile acid malabsorption, and/or small intestinal overgrowth. Further interpretation of potential markers across studies is challenging due to differences in study design, inclusion criteria, improvements in experimental and computational methods over time and, critically, confounding by sampling of different tissues and cellular heterogeneity. These challenges may explain why we were unable to replicate here the previous associations with oncostatin M [*OSM*]^9,10^ and triggering receptor expressed on myeloid cells [*TREM-1*]^11,12^ identified as potential biomarkers predicting non-response to anti-TNF treatment. Our data argue against the presence of a single epigenetic biomarker in whole blood which has clinical utility.

Our observations that higher DNA methylation epigenetic smoking score and smoking status, and epigenetic age of participants and chronological age, were highly positively correlated internally, validate our DNA methylation processing and quality control methods, supporting subsequent findings against clinical outcomes. Interestingly, increase in smoking score was observed in all groups regardless of smoking status over time, but was significantly less in former smokers compared with never smokers. Prior longitudinal studies of DNA methylation changes following smoking cessation have reported conflicting results,^37,38^ although varying follow-up times and the study of different populations make it difficult to compare them across studies. Whether the DNA methylation changes following smoking cessation have an impact on anti-TNF drug concentration or outcomes in patients with Crohn’s disease requires further investigation.

### 4.3. Limitations and generalisability

We acknowledge some important limitations of our work. First, our outcome data could be strengthened with endoscopic outcomes. However, we observed a significant association between clinical outcomes at week 14 and week 54 and faecal calprotectin, which has been shown to closely correlate with endoscopic findings. Second, we measured DNA methylation from whole blood, which is likely to be confounded by differences in individual cell proportions. Although we included derived cell proportions as a covariate in our statistical models, this is unlikely to fully control for cellular changes that may be better controlled for by expanded panels of blood cell types or single-cell analyses. Whether similar changes also occur in the target tissues in the small and large intestine is unknown. Third, our findings should be validated in an independent cohort prior to translation into clinical practice.

The PANTS study recruited patients from across the UK, and we believe our findings will be generalisable to patients with Crohn’s disease treated with an anti-TNF across other western populations. Further work is required to determine if these findings are found in other non-western populations, and indeed in other populations of patients with IBD such as those with ulcerative colitis and in non-IBD patients treated with an anti-TNF.

In conclusion, baseline DNA methylation profiles may be used as a predictor for anti-TNF drug concentration at week 14 to identify patients who may benefit from dose optimisation at the outset of anti-TNF therapy.

## Supplementary Data

Supplementary data are available at *ECCO-JCC* online.

jjad133_suppl_Supplementary_Material

## Data Availability

Individual participant de-identified data that underlie the results reported in this article will be available immediately after publication for a period of 5 years. The data will be made available to investigators whose proposed use of the data has been approved by an independent review committee. Analyses will be restricted to the aims in the approved proposal. Proposals should be directed to [tariq.ahmad1@nhs.net]. To gain access, data requesters will need to sign a data access agreement.
